# Endothelial progenitor cells and integrins: adhesive needs

**DOI:** 10.1186/1755-1536-5-4

**Published:** 2012-03-12

**Authors:** Francisco Caiado, Sérgio Dias

**Affiliations:** 1 Angiogenesis Laboratory, CIPM, Instituto Português de Oncologia Francisco Gentil, EPE, Lisboa, Portugal; 2 Instituto Gulbenkian Ciencia, Oeiras, Portugal; 3 CEDOC, Faculdade de Ciências Médicas, Lisbon, Portugal

## Abstract

In the last decade there have been multiple studies concerning the contribution of endothelial progenitor cells (EPCs) to new vessel formation in different physiological and pathological settings. The process by which EPCs contribute to new vessel formation in adults is termed postnatal vasculogenesis and occurs via four inter-related steps. They must respond to chemoattractant signals and mobilize from the bone marrow to the peripheral blood; home in on sites of new vessel formation; invade and migrate at the same sites; and differentiate into mature endothelial cells (ECs) and/or regulate pre-existing ECs via paracrine or juxtacrine signals. During these four steps, EPCs interact with different physiological compartments, namely bone marrow, peripheral blood, blood vessels and homing tissues. The success of each step depends on the ability of EPCs to interact, adapt and respond to multiple molecular cues. The present review summarizes the interactions between integrins expressed by EPCs and their ligands: extracellular matrix components and cell surface proteins present at sites of postnatal vasculogenesis. The data summarized here indicate that integrins represent a major molecular determinant of EPC function, with different integrin subunits regulating different steps of EPC biology. Specifically, integrin α4β1 is a key regulator of EPC retention and/or mobilization from the bone marrow, while integrins α5β1, α6β1, αvβ3 and αvβ5 are major determinants of EPC homing, invasion, differentiation and paracrine factor production. β2 integrins are the major regulators of EPC transendothelial migration. The relevance of integrins in EPC biology is also demonstrated by many studies that use extracellular matrix-based scaffolds as a clinical tool to improve the vasculogenic functions of EPCs. We propose that targeted and tissue-specific manipulation of EPC integrin-mediated interactions may be crucial to further improve the usage of this cell population as a relevant clinical agent.

## Review

### Postnatal vasculogenesis and endothelial progenitor cells

The cardiovascular system is the first functional organ system to develop in the vertebrate embryo and is required for embryonic survival to regulate multiple homeostatic functions in the developing embryo [1]. New blood vessel formation (neovascularization) is an essential mechanism determining the formation, but also the maintenance, of the cardiovascular system. It is thought to depend mainly on two processes, angiogenesis and vasculogenesis.

Angiogenesis is the process by which new vessels are formed by the activation, proliferation and migration of endothelial cells (ECs). Vasculogenesis is defined as the process by which new vessels are generated, by the migration and differentiation of vascular endothelial growth factor receptor 2 positive (VEGFR-2+) mesodermal precursors, termed angioblasts and/or hemangioblasts, into ECs that coalesce to form a primary vascular plexus during embryonic development [2].

The existence of an equivalent process during adulthood - postnatal vasculogenesis - has been intriguing vascular and hematologic researchers since the early 20th century, when the first studies describing blood vessel formation from peripheral blood (PB) and bone-marrow (BM) mononuclear cells were published. These studies suggested the existence of a population of cells in the PB and/or BM capable of generating ECs when cultured under specific conditions [3-5]. However, it was only in 1997 that Asahara *et al. *[6] isolated and characterized CD34+ or VEGFR-2+ cell populations for the first time, derived from PB capable of differentiating into ECs *in vitro *when plated on fibronectin (FN) and exposed to angiogenic growth factor stimuli, namely vascular endothelial growth factor (VEGF). Accordingly, using an *in vivo *animal model of hind limb ischemia, these authors showed that CD34+ and VEGFR-2+ cells were incorporated into newly formed vessels and acquired the expression of EC antigens. These cells where thus termed endothelial progenitor cells (EPCs) [6]. In accordance, Shi Q et al [7] reported the existence of 'circulating bone marrow-derived endothelial progenitor cells' in the adult and showed that these cells were derived from BM. Circulating BM-derived EPCs were defined as a subset of CD34+ hematopoietic stem cells with the ability to differentiate into the endothelial lineage and express endothelial marker such as von Willebrand Factor (vWF) and incorporate acetylated Low Density Lipoprotein (Ac-LDL). Most convincingly, these authors showed that bone marrow-transplanted genetically tagged cells contributed to the endothelialization of a Dacron graft placed on the descending thoracic aorta in dogs. Later, in 2000, Peichev *et al. *added to the molecular definition of human EPCs, showing that these express CD133+ (prominin), CD34+ and VEGFR-2+, and are present in adult mobilized PB, cord blood and also fetal livers. This pioneer study proposed that CD133, together with other endothelial markers, including VEGFR2 and CD34, could be used to distinguish EPCs from mature ECs and also from other tissue stem cells [8]. The proof of existence for EPCs and the demonstration of their ability to differentiate into mature ECs and incorporate blood vessels was a key finding to support the existence of postnatal vasculogenesis.

The biological significance of EPCs is supported by accumulating evidence from the last decade showing that this cell population contributes in a quantitative manner to postnatal vasculogenesis in multiple conditions These include physiological, such as neonatal tissue growth [9,10], and pathological conditions, such as peripheral vascular disease [11], myocardial and limb ischemia [12,13], stroke [14], tissue regeneration [15-18], retinopathy [19], artherosclerosis [20] and tumor growth [21-24]. Given their significant biological contribution in different types of vascular pathologies, EPCs and their biology have been under intense investigation for the last decade. Most studies have focused on the molecular definition of EPCs, on the molecular mechanisms regulating EPC function, the quantitative determination of EPC contribution to physiological and/or pathological postnatal vasculogenesis and, more recently, on the clinical and therapeutic applications of this cell population.

In the next sections of this review we will update and resume some of these aspects with a major focus on the importance of integrin-mediated interactions (adhesion) on EPC biology and function.

#### Molecular definition(s) of endothelial progenitor cells

A consensus as to the markers that define EPCs has not been reached; different authors use various combinations of surface and functional markers to isolate and study EPCs in different contexts. In the context of this review, EPCs will be defined as BM-, PB-, cord blood- or fetal liver-derived nonendothelial cells that have stemness properties and markers (CD133+, CD34+, c-Kit+/Sca-1+); are capable of clonal expansion; and are able to differentiate into adherent ECs, acquiring endothelial properties and markers (CD146+, CD31+, CD105+, vWF+, tyrosine kinase with immunoglobulin-like and EGF-like domains-2+, vascular endothelial cadherin+ and VEGFR-2+) [25-27]. A more comprehensive definition of EPC markers is beyond the scope of this review.

#### Endothelial progenitor cell biology and function during postnatal vasculogenesis

Since EPCs were first described there have been numerous studies concerning their biology and function. In the last decade, it has been established that, in order to exert their 'vascular function', EPCs have to accomplish four distinct but interrelated steps. They must respond to chemoattractant signals and mobilize from the BM to the PB; home in on sites of vascular remodeling, repair and angiogenesis; invade and migrate at the same sites; and differentiate into mature ECs and/or regulate pre-existing ECs via paracrine or juxtacrine signals [28].

**Figure 1:**
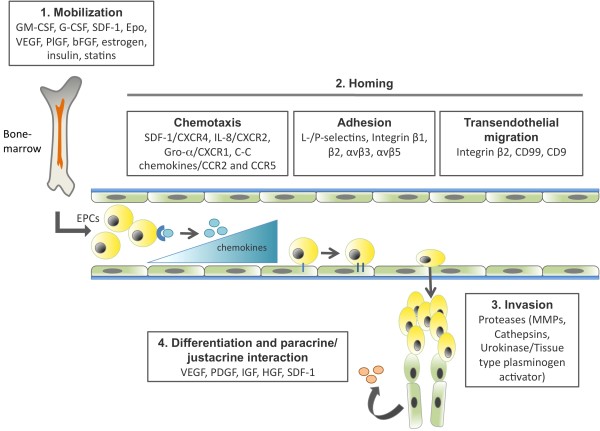
**Molecular mechanism regulating the multiple steps of endothelial progenitor cell biology during postnatal vasculogenesis**. Recruitment and incorporation of EPCs into angiogenic sites requires a coordinated multistep process including mobilization, chemoattraction, adhesion, endothelial transmigration, migration, tissue invasion, in situ differentiation and paracrine and/or juxtacrine factor production. The major molecular mechanisms that have been implicated in the distinct steps of EPC biology are indicated. Figure adapted from Fusenig N, Marmé D (eds): *Tumor Angiogenesis: Basic Mechanisms and Cancer Therapy*. Springer; 2008. Chapter 17 with modifications [126].

Some of the major molecular regulators that determine these four steps have been identified (Figure 1). In normal conditions, EPCs reside within a stem cell niche in the BM characterized by low oxygen tension [29] and high levels of stromal cell-derived factor-1 (SDF-1), a potent chemoattractant for EPCs that binds via the receptor CXC chemokine receptor type 4 (CXCR4) [30]. EPCs are mobilized from the BM in response to peripheral tissue hypoxia and trauma, which cause the production and release of EPC mobilizing factors such as granulocyte-monocyte colony stimulating factor, granulocyte colony stimulating factor, VEGF, basic fibroblast growth factor, placental growth factor, erythropoietin or SDF-1 to a concentration greater than that in the BM [28]. These factor act via the phosphoinositide 3-kinase/protein kinase B (Akt) pathway to activate endothelial nitric oxide synthase, leading to an increased production of nitric oxide, which regulates the enzymatic activity of matrix metalloproteinases (MMPs) [31,32]. In particular, activated MMP-9 leads to the release of soluble kit ligand from EPCs in the BM, allowing the cells to move out to the peripheral circulation [33].

Once in circulation, EPCs home in on activated tissues in response to chemokine gradients that are formed in the tissue regions undergoing active remodeling. The major chemokines and respective receptors that regulate EPC activation and homing are SDF-1 and CXCR4; IL-8 and CXCR2; growth regulated oncogene-α and CXCR1; and C-C chemokine and chemokine (C-C motif) receptors 2 and 5 [34-39]. Upon interaction with tissue-specific chemokines, EPCs become activated and initiate integrin-mediated adhesion to endothelial vascular cells and consequently transendothelial migration into sites of vascular and tissue remodeling. The major integrins and respective ligands that mediate these steps of EPC function will be discussed in detail in the present review.

Once circulating EPCs have crossed the endothelial monolayer, they need to migrate through the blood vessel basement membrane and through the interstitial extracellular matrix (ECM) in order to arrive at the specific niches where they can exert their functions. EPC invasion depends to a great extent on the activity of extracellular proteases that breakdown and remodel ECM components at the vessel basement membrane and also in the interstitial space. The major extracellular proteases involved in EPC invasion are members of the MMP family (in particular MMP-9 [40]), members of the cathepsin family (cathepsin L [41]) and also the serine protease urokinase-type plasminogen activator and tissue-type plasminogen activator [42].

When the EPCs arrive at sites of vessel remodeling they will contribute to new vessel formation and remodeling. The mechanisms that contribute to the functional activity of EPCs are still under investigation. However, functional activity of EPCs depends mainly on two processes: differentiation into mature ECs and direct incorporation into neovessels and/or production of paracrine and/or juxtacrine signals that promote interactions with pre-existing ECs and other cell types. The differentiation of EPCs into ECs is a very complex process that can be subdivided into three steps [43]. First, there is integrin-mediated adhesion to ECM components. The direct interaction between integrin α5β1 and FN in particular have been shown to be essential in the initial steps of EPC differentiation [44]. Second, there is growth factor-induced proliferation and survival: the VEGF family of growth factors and also the angiopoietin-tyrosine kinase with immunoglobulin-like and EGF-like domains 1 receptor pathway [45] have been implicated in the regulation of EPC proliferation and survival. Third, the maturation and acquisition of an endothelial phenotype is an essential step in EPC biology and depends mainly on the regulation of the transcription factor HoxA, which is transcriptionally regulated by histone deacetylases. Accordingly, HoxA9 has been shown to regulate the expression of the endothelial genes for endothelial nitric oxide synthase, VEGFR-2 and vascular endothelial cadherin [46]. Besides differentiating into ECs, EPCs have been shown to produce multiple paracrine factors such as VEGF, SDF-1, insulin-like growth factor 1, monocyte chemotactic protein 1 (MCP-1), macrophage inflammatory protein 1α and platelet derived growth factor [47,48]. Altogether these factors can act on different cell types leading to an overall response that promotes angiogenesis and tissue regeneration.

### Integrin-extracellular matrix interactions in endothelial progenitor cells biology

Throughout the main steps that regulate EPC function - mobilization, homing, invasion and differentiation and/or paracrine factor production - EPCs interact with different physiological compartments, namely BM, PB, blood vessels and homing tissues. Therefore what determines the success of each step is the ability of EPCs to interact, adapt and respond to multiple molecular cues present in each compartment. Considering this, although some of the molecular pathways regulating the multiple steps of EPC biology have been addressed (Figure 1), in the present review we will summarize the known contributions of structural and/or morphogenic signals to EPC biology, in particular the interaction between integrins expressed by EPCs and their ligands, ECM components and cell surface proteins present at sites of vascular repair/remodeling. We propose that targeted manipulation of integrin-ECM interactions in EPCs and at sites of vascular remodeling may contribute to the improvement of EPC-mediated vascular repair and function.

The ECM is a non-cellular component present within all tissues and organs that provides not only an essential physical scaffold for the cellular constituents but also initiates crucial biochemical and biomechanical cues that determine cell differentiation, proliferation, survival, polarity and migration. It thus plays an essential role in tissue morphogenesis, differentiation and homeostasis [49]. The ECM is mainly composed of two main classes of macromolecules: proteoglycans and fibrous proteins [50,51]. The main fibrous ECM proteins are collagens, elastins, FNs and laminins. Proteoglycans (namely perlecan) fill the majority of the extracellular interstitial space within the tissue in the form of a hydrated gelatin. The direct effect of ECM components in cell behavior derives mainly from two ECM properties: their ability to bind directly to their cellular receptors, integrins and discoidin domain tyrosine kinase receptors - which in turn are signal transduction receptors; and their ability to bind and present growth factors as organized solid-phase [49]. As mentioned earlier, in the present review we will focus mainly on the contribution of ECM-integrin interactions on EPC biology.

Integrins are a family of non-covalently associated heterodimeric transmembrane glycoprotein adhesion proteins which mediate cell-ECM and cell-cell interactions. Integrins are responsible for cellular tissue architecture and also function as signal transducers regulating survival, proliferation, differentiation and migratory signaling pathways. Each integrin is composed of two subunits: one α- (around 800 amino acids) and one β-subunit (approximately 1,000 amino acids). In higher vertebrates, eighteen α- and eight β-subunits combine to form more than 24 different integrin heterodimers. Heterodimer composition confers ligand specificity, with most integrins recognizing several ECM proteins that in turn bind to more than one integrin [52].

#### Integrin expression in endothelial progenitor cells

In agreement with multiple studies concerning the expression of integrin subunits on EPCs, we can define an integrin expression profile on EPCs. Accordingly, EPCs express integrin subunits α1, α2, α3, α4, α5, α6, α9, αv, β1, β2, β3, β5 and β7 [43,44,53-61]. Despite the expression of so many different integrin subunits by EPCs, it is apparent that some of these integrins can in fact be activated and/or upregulated at specific steps of EPC biology as reflex to the multiple molecular cues (tissue specific ECM and soluble factors) that may be present. This suggests that the integrin expression profile of EPCs is in fact a dynamic aspect of EPC biology reflecting the adaptation of these cells to different conditions. In the next sections we will summarize the reported functions of each integrin subunit in EPC biology (Table 1).

**Table 1:** Overview of the major integrin sub-units and respective ligands involved in endothelial progenitor cell biology.

Integrin	Main ligand	Role on EPC biology	References
α4β1	FN; VCAM-1	Bone marrow retention and/or mobilization; homing and/or adhesion to angiogenic sites	[63,72]
α5β1	FN	Homing and/or adhesion to angiogenic sites; invasion and migration; differentiation and paracrine/juxtacrine interaction	[44,69,83,96-98]
α6β1	Laminin	Homing and/or adhesion to angiogenic sites; invasion	[56,87]
β2 integrins	Fibrin(ogen); ICAM-1/2	Homing and/or adhesion to angiogenic sites; transendothelial migration	[72-74,90,91]
αvβ3	RGD	Homing and/or adhesion to angiogenic sites; bone marrow retention and/or mobilization (β3 subunit)	[69,88,98]
αvβ5	RGD	Homing/adhesion to angiogenic sites; differentiation and paracrine and/or juxtacrine interaction (β5 subunit)	[88,89,98,99]

EPC: endothelial progenitor cell; FN: fibronectin; ICAM-1/2: intercellular adhesion molecule 1/2; RGD: arginine-glycine-aspartate motif; VCAM-1: vascular cell adhesion molecule 1.

### Integrin-mediated endothelial progenitor cell mobilization from the bone marrow - integrin subunits α4 and β3

The main integrins regulating the mobilization of EPCs from the BM microenvironment are the α4 integrins. These have been proven essential for embryogenesis, hematopoiesis, lymphocyte homing and the recruitment of leukocytes to sites of inflammation [62]. The α4 integrins, α4β1 and α4β7 are most prominent on mononuclear leukocytes, but can also be expressed in neutrophils, hematopoietic stem cells and EPCs [63,64]. α4β1 mediates cell adhesion to vascular cell adhesion molecule-1 (VCAM-1) and to an alternatively spliced form of the extracellular matrix protein, FN [65]. α4β7 is important in lymphocyte homing to mucosal tissue by adhering to the gut homing receptor mucosa addressing cell adhesion molecule and it also binds to VCAM-1 and FN [66-68]. Concerning their role on EPC mobilization, it has been shown that *in vitro *usage of anti-α4 integrin antibody blocks and competes with the adhesive interactions between BM-derived EPCs and immobilized VCAM-1, FN (to a smaller extent) or BM stromal cells. Moreover, systemic administration of anti-α4 integrin antibody or conditional knockout of α4 integrin in the BM significantly increases the number of circulating EPCs, suggesting that this integrin is essential for retaining EPCs in the BM [63]. Interestingly, after ischemic injury, integrin α4 blockade results in an increase in the number of BM-derived EPCs present in the neovasculature at the ischemic tissue and augments the recovery of blood flow and tissue preservation. Altogether, these authors establish that α4 integrin plays an important role in BM-derived EPC mobilization and that functional disruption of α4 integrin-mediated EPC retention in the BM causes a shift toward a distribution of EPCs into circulation that favors neovascularization.

Another integrin subunit that has been implicated in the retention of EPCs in the BM is the integrin β3. These integrins have essential roles in angiogenesis, hemostasis and in bone remodeling, being essentially expressed on ECs, platelets, osteoclasts and hematopoietic cells [52]. Concerning EPC mobilization, a recent paper by Watson *et al. *shows that β3 integrins are necessary for the retention of EPCs in the BM, since wild-type mice receiving β3 integrin-null BM show increased EPCs mobilization to the PB and consequent increased tumor vascularization. However, a high proportion of the vessels are nonfunctional and therefore do not enhance tumor growth, further suggesting that integrin β3 might affect other EPC properties besides BM mobilization [69].

### Integrin-mediated endothelial progenitor cells homing to sites of vascular remodeling, repair and angiogenesis

After being mobilized from the BM into the PB, EPCs become a small percentage of the circulating cells along with other hematopoietic populations. Considering this, EPCs must be specifically responsive to signals present at injury and remodeling ECs as opposed to signals present in normal and quiescent ECs. Recognizing such signals enables EPCs to home to sites of vessel injury and remodeling, where they become activated by local chemokines and cytokines, adhere to activated ECs or ECM components and incorporate and differentiate into newly formed vessels, or act via paracrine and/or juxtacrine interactions on pre-existing ECs. Direct evidence of EPC homing and incorporation into neovasculature has been addressed by Vajkoczy *et al. *using intravital fluorescence videomicroscopy and murine embryonic EPCs in a tumor model. According to this study embryonic EPC homing to tumor vasculature occurs mainly by sticking (active adhesion to tumor vasculature without affecting blood flow) and not by plugging tumor blood vessels (size restrictions or dead-end vascular sprouts), contrary to what happens in normal vasculature [70]. This data suggests that EPC homing is an active process involving direct interaction between molecular targets expressed on homing tissues and adhesion molecules, namely integrins, expressed by EPCs.

#### β2 integrins in endothelial progenitor cell homing

There are numerous studies reporting the role of integrins during EPC homing to sites of neoangiogenesis. One of the major integrin subunits regulating EPC homing to active angiogenic sites is the β2 integrin. β2 integrins are described as leukocyte specific receptors, recognizing essentially multiple members of the intercellular adhesion molecule (ICAM) family and polysaccharides [71]. These integrins are essential for the regulation of hematopoiesis, leukocyte recruitment and inflammatory cells [52]. Despite the specificity of integrin β2 expression in leukocytes, many studies have described its expression in EPCs [61,71-73]. *In vitro *adhesion studies show that β2 integrins mediate the adhesion of adult PB-derived EPCs to pre-activated EC monolayers and also to ICAM-1 and fibrinogen. Additionally, the same study revealed that β2 integrins and its activation status play an essential role not only in the homing of BM-derived EPCs to ischemic tissues but also in the neovascularization capacity of these cells *in vivo*. Further supporting the role of integrin β2 and its activation on EPC homing is the fact that the addition of pharmacological activators of exchange protein directly activated by cyclic AMP (a nucleotide exchange protein for ras-related protein 1 that activates integrin conformation) to EPCs increases β2-integrin-dependent adhesion to ICAM-1, migration on fibrinogen and, consequently, the homing and neovascularization-promoting capacity of intravenously injected EPCs [74].

#### β1 integrins in endothelial progenitor cell homing

In spite of the described role of β2 integrins on EPC homing, multiple studies have shown that its inhibition leads only to a partial inhibition of EPC homing, suggesting that other integrins may also regulate this essential step of EPC biology. One of the major integrin families regulating EPC homing to angiogenic sites is the β1 integrins.

In particular integrin α4β1 is highly expressed in EPCs and has been shown to mediate *in vitro *adhesion of EPCs to pre-activated EC monolayers, specifically to VCAM-1 and cellular FN [63]. Furthermore, in *in vivo *models of breast cancer, inhibition of α4β1 integrin significantly blocks homing of BM-derived EPCs cells to tumor neovessels expressing its ligands FN and VCAM-1 [72]. Interestingly, as mentioned before, α4β1 blockade can in fact mobilize EPCs from the BM and increase their number in the PB, thus indicating that targeting this integrin can have a double effect, increasing EPC mobilization but decreasing their homing to angiogenic sites.

Besides recognizing molecular homing signals expressed by activated ECs, EPCs can recognize and adhere directly to exposed ECM components. α5β1 integrin is an FN receptor, which binds to the arginine-glycine-aspartic acid (RGD) motif region of FN [75]. Studies using various cell culture systems have suggested that α5β1 is involved in many cellular processes including cell proliferation, oncogenic transformation, assembly of FN-rich extracellular matrices, cell migration, regulation of gene expression, wound healing, T-cell activation, angiogenesis and embryogenesis - where it regulates mesodermal formation and function [76-80]. Integrin α5β1 is also highly expressed in EPCs and is directly involved in the homing of EPCs to denuded vessels, where its ligand FN rapidly accumulates [81]. Accordingly, treatment of EPCs with statins increases the expression levels of integrin α5β1, consequently increasing EPC homing to vascular injury sites and thus promoting re- endothelialization of these sites [59].

In agreement with the above, a recent report describes the direct interaction between EPCs integrin α5β1 and FN under laminar shear stress, suggesting that EPC adhesion to exposed FN on blood vessels can occur under physiological levels of shear stress [82]. The role of α5β1 in EPC homing is further supported in models of lung vascular injury, where these integrins, together with α4β1 integrin, mediate the homing and adhesion of EPCs to damaged pulmonary capillaries, further contributing to the repair of the endothelial barrier and preventing lung vascular damage [83].

Another β1 integrin associated with EPC homing to sites of neovascularization is the integrin α6β1. α6β1 integrin is a laminin-binding integrin that is essential for skin homeostasis [84], regulates endothelial tube formation [85]and also contributes to hematopoietic stem cell homing to the BM [86]. Concerning EPCs, a recent work by Bouvard *et al. *has shown that integrin α6β1 is directly regulated by VEGF and basic fibroblast growth factor and is necessary for EPC homing to ischemic skeletal muscle *in vivo *and to EPC adhesion to basement membrane components *in vitro*. According to these authors, vessel obstruction leads to reduced oxygen supply and consequent death and detachment of the ECs lining the walls, exposing the underlying basement membrane components (namely laminins). Exposed laminin may then function as a specific homing signal for EPCs expressing integrin α6β1, thus directing EPCs to sites of vascular injury where these cells can restore vascular integrity [56,87].

#### αv integrin in endothelial progenitor cell homing

Integrins αv (namely αvβ3- and αvβ5-integrins) also play a role in EPC homing to sites of vascular repair. αv integrins are expressed on almost all the cells originating from the mesenchyme and mediate many biologic events, such as migration of vascular smooth muscle cells (SMCs), adhesion of osteoclasts to the bone matrix and angiogenesis. αv integrins are known to bind different ligands, including vitronectin, FN, osteopontin, fibrinogen and vWF, by interacting with the RGD motif [52]. Interestingly, EPC adhesion to denuded vessels appears to be also mediated by αvβ3- and αvβ5-integrins, since inhibition of αvβ3- and αvβ5-integrins with cyclic RGD peptides blocks EPC-mediated re-endothelialization of denuded arteries [88]. In agreement, integrin αvβ5 has been described as essential to the adhesion of EPCs to differentiated endothelial cells [89].

### Integrin-mediated endothelial progenitor cells transendothelial migration

Once EPCs adhere at specific homing sites they need to migrate through the endothelial monolayer and invade the underlying tissue. Concerning EPCs, very little is known in relation to integrins mediating transendothelial migration. In human adult PB-derived EPCs, transendothelial migration appears to be mediated mainly by β2 integrins and depends on MCP-1 and VEGF [90]. This is further supported by a study showing that increased expression of ICAM-1 in ECs, via Akt activation, increases EPC homing and transendothelial migration in *in vitro *assays [91].

### Integrin-mediated endothelial progenitor cell invasion and migration

As EPCs cross the endothelial monolayer, they need to migrate through the blood vessel basement membrane and through the interstitial ECM in order to arrive at the specific niches where they can exert their functions. These processes require multiple cell-ECM interactions and depend mainly on the integrin-ECM interactions and on extracellular proteases-ECM interactions. The essential role of the ECM as a provider of biochemical and biophysical cues that regulate EPCs cellular behavior is addressed in a recent report by Hanjaya-Putra *et al*., where the authors show that the ECM stiffness modulates EPC invasion. In detail, EPCs can sense matrix stiffness through signaling cascades downstream of integrin ligation leading to the activation of the Rho guanosine triphosphate hydrolase, cell division control protein 42 homolog. Accordingly, if the matrix stiffness is high, EPCs upregulate the production of extracellular proteases that allow both matrix degradation and EPC migration [92]. This study revealed that both integrins and extracellular proteases are essential to modulate EPC invasion and migration along the ECM. The contribution of integrins to EPC invasion and migration is further supported by Wijelath *et al. *These authors show that integrin α5β1 mediates EPC migration on FN towards a VEGF gradient, *in vitro *[44]. In another study, integrin α6β1 is described as an essential integrin regulating EPC adhesion and migration towards VEGF, in a phosphatidylinositol 3-kinase/Akt pathway dependent way. Furthermore, the same authors show that α6β1 integrin mediates EPC invasion and consequent tube formation *in vitro *and *in vivo*, being essential for EPC-mediated collateral vessel formation in ischemic hind limb mouse models [56,87].

### Role of integrin-endothelial progenitor cell interactions on endothelial progenitor cell differentiation

The functional activity of EPCs depends mainly on two interdependent processes: differentiation into mature ECs and direct incorporation into neovessels and/or production of paracrine and/or juxtacrine signals that promote interactions with pre-existing ECs and other cell types. Concerning EPC differentiation into EC, a study by our laboratory has shed some light onto the molecular pathways that modulate the different steps of EPC differentiation. We defined a global gene-expression profile of cord blood-EPC (CD133+, CD34+ and kinase insert domain receptor +) during the process of endothelial differentiation *in vitro*. The gene profile data of EPC differentiation reveals a strict temporal regulation of endothelial differentiation, which can be divided into three sequential and distinct stages: integrin-mediated adhesion to specific ECM components; growth factor-induced proliferation and survival; and maturation and functional acquisition of EC properties [43].

EPC adhesion to the ECM is an essential step during differentiation, allowing cells to attach onto a substrate and to acquire proliferative and survival signals from the underlying matrix. Moreover, direct interaction between integrins and ECM can regulate EPC paracrine factor production. Interestingly, the integrin expression profile in EPCs is variable throughout endothelial differentiation, further suggesting that integrin-ECM interactions can mediate different aspects of the endothelial differentiation process. In detail, integrin α9 is only expressed in undifferentiated EPCs (day 0 of differentiation), while β5 and β7 are only expressed in differentiating EPCs (day 13). On the other hand, integrin subunits α4, α5 and αv are expressed throughout EPC differentiation. All the non-variable integrin subunits are FN-binding integrins, further pointing out that direct interaction of EPCs with FN is essential during endothelial differentiation. In fact, FN has been shown to be essential for vasculogenesis during embryonic development, as well as its receptor integrin α5β1. Accordingly, genes for FN and integrin subunit α5 are among the 12 genes considered critical for vasculogenesis since knockout mice for these genes are embryonic lethal with major vascular defects [93-95]. Concerning EPCs, early work by Asahara *et al. *revealed for the first time that these cells show higher adhesion and endothelial differentiation when plated on FN compared to collagen [6]. More recently, the role of the FN-integrin α5β1 interaction in EPC differentiation has been addressed. FN is described as a major regulator of EPC differentiation since it promotes VEGF-induced differentiation of EPCs into ECs via specific binding to integrin α5β [44]. Although the precise mechanism by which FN and VEGF synergy regulates EPC differentiation is not clear, it is possible that the formation of FN/VEGF complexes by direct interactions of VEGF with FN heparin-II domain [96] leads firstly to the physical association between VEGFR-2 and α5β1 [97] and consequently to the activation of downstream pathways that may potentiate EPC endothelial differentiation.

The contribution of integrin-ECM interactions in EPC differentiation is further demonstrated in a recent study showing that vascular endothelial growth inhibitor, a known anti-angiogenic cytokine, impairs EPC adhesion and differentiation on both FN and vitronectin by downregulating the expression of integrin subunits α5 and αv, further suggesting the importance of both integrins during EPC differentiation [98]. Besides differentiating into ECs, EPCs can produce multiple paracrine factors that in turn can act on other cell types influencing multiple cellular processes. Interestingly, there is some data suggesting that integrin-ECM interactions can influence EPC paracrine factor production. For instance, integrin β5 regulates EPCs paracrine factor production. Overexpression of integrin β5 in EPCs results in integrin αvβ5 phosphorylation, activation of proto-oncogene tyrosine-protein kinase and activation of transcription factor STAT3, which in turn induce expression of the pro-angiogenic factors IL-8 and MCP-1 [99].

Recent investigation by Barsotti *et al. *shows that EPCs plated on different matrices produce different amounts of paracrine factors. For instance, when plated on fibrin, EPCs expressed increased levels of multiple cytokines, namely IL-16, platelet derived growth factor-BB, SDF-1, hepatocyte growth factor (HGF), Interferon gamma-induced protein 10 (IP-10) and monokine induced by gamma interferon (MIG). This furthers suggests that the paracrine factor production by EPCs is also regulated by the integrin-ECM interactions [100]. Additionally, recent data collected from our group also suggests that EPCs grown on different ECM components produce different levels of paracrine factors. We observed that EPCs grown or gelatin, FN or on fibrin proteolytic fragment E (FbnE) express different levels of multiple paracrine factors. They showed higher expression of VEGF-A, transforming growth factor β1, SDF-1, IL-8 and macrophage inflammatory protein-1α, however the precise mechanism downstream of the interaction between integrin α5β1 and FbnE was not established [54].

### Endothelial progenitor cells as therapeutic agents - how to improve endothelial progenitor cell function *in vivo*: extracellular matrix scaffolds

Since EPCs where first isolated, many *in vitro *and *in vivo *preclinical studies have created great expectation for their wide use in clinical practice. In fact, to date, a dozen of complete interventional clinical trials concerning the application of PB or BM-derived EPCs in cardiovascular diseases have been published (Table 2) while many others are ongoing [111]. Despite the success of the application of EPCs as therapeutic agents in cardiovascular disease, there are some limitations to the interpretation of these clinical trials - namely the lack of appropriate controls, randomization and blinding. Other limitations are the use of distinct EPC populations, routes of delivery used and timing of cell delivery, all variables rendering cross-sectional comparison difficult [111]. Concerning the routes of delivery, studies have shown that the therapeutic capabilities of EPCs upon *in vivo *systemic transplantation were insignificant, because of their poor bio-distribution and low cell survival [112,113]. To avoid these drawbacks, several studies have attempted to inoculate EPCs directly at sites of vascular repair and healing, including infracted areas of the heart. Compared to systemic injection, this approach led to significantly greater, but still marginal, numbers of injected cells that survive in the infarct area and eventually contribute to tissue regeneration [114]. To overcome these limitations of conventional transplantation methods, there has been intense development of scaffolds that promote *in situ *cell retention and avoid non-specific cell homing. Considering the essential role of integrin-ECM interactions in EPC biology there has been a major effort to develop scaffolds constituted by biologically or artificially derived ECM that provide EPCs with ECM survival and proliferative and differentiating signals, rendering EPC *in vivo *application more efficient.

**Table 2:** Published interventional clinical trials using endothelial progenitor cells.

Disease	Patient numbers	Intervention	Description	Results	Reference
Chronic ischemic heart disease	121	Transcoronary transplantation of bone marrow-derived progenitor cells	Intracoronary infusion in patients with chronic ischemic heart disease	Reduced serum levels of heart failure markers; reduced mortality	[101]
Idiopathic pulmonary arterial hypertension	33	Transplantation of autologous EPCs (differentiated from peripheral blood mononuclear cells)	Test safety, feasibility, and initial clinical outcome of intravenous infusion of autologous EPCs in patients with idiopathic pulmonary arterial hypertension	Feasible and safe infusion of autologous EPCs is beneficial to exercise capacity and pulmonary hemodynamics in patients with idiopathic pulmonary arterial hypertension	[102]
Chronic ischemic heart disease	75	Intracoronary infusion of peripheral blood-EPCs and bone marrow-derived progenitor cells	Effect of intracoronary EPC infusion on the left ventricular contractile function	Feasible and safe transplantation of bone marrow-derived progenitor cells is associated with moderate but significant improvement in the left ventricular ejection fraction after three months	[103]
Acute myocardial infarction	26	Intracoronary injection of EPCs (differentiated from peripheral blood mononuclear cells)	Effect of intracoronary EPC infusion on coronary vasomotion and left ventricular function in patients after recanalization of chronic coronary total occlusion	Increased left ventricular ejection fraction, coronary flow reserve; reduction in infarct size	[104]
Acute myocardial infarction	11	Transcoronary transplantation of bone marrow-derived EPCs and mesenchymal stem cells	Effect of transcoronary transplantation of EPCs and mesenchymal stem cells on myocardial contractility and tissue regeneration	Reduction in infarct size	[105]
Acute myocardial infarction	59	Intracoronary infusion of peripheral blood EPCs and bone marrow-derived progenitor cells	Effect of intracoronary EPC infusion on left ventricular function, infarct size and reactive hypertrophy	Increased left ventricular ejection fraction, no reactive hypertrophy; Reduction in infarct size	[106]
Critical limb ischemia	28	Intramuscular injections of peripheral blood granulocyte colony stimulating factor mobilized CD34+ CD133+ EPCs	Effect of intramuscular infusion on and limb salvage rate for amputation at 12 months	Implantation of EPCs in critical limb ischemia is a safe alternative, improves tissue perfusion, and obtains high amputation-free rates	[107]
Acute myocardial infarction	366	EPC capture stent	Safety and efficacy of EPC capture stents in the acute myocardial infarction settings	EPC stent is safe; At two-year follow-up, the EPC group showed favorable target vessel revascularizations rate and stent thrombosis remained a low-event occurrence	[108]
ST elevation acute myocardial infarction	100	EPC capture stent	Safety and efficacy of EPC capture stents in the ST elevation acute myocardial infarction settings	The study does not support the use of EPC capture stents with short duration dual antiplatelet therapy in patients with ST elevation acute myocardial infarction	[109]
Refractory angina	167	Intramyocardial injections of autologous CD34+ cells	Effect of intramyocardial injections of autologous CD34+ cells on refractory angina patient	Intramyocardial injections of autologous CD34+ cells improves angina frequency and exercise tolerance	[110]

EPC: endothelial progenitor cell.

Biological scaffolds are composed of naturally occurring polymers present in the ECM, namely proteins and polysaccharides. The main proteins used for scaffold composition are collagens, fibrin and gelatin. Collagen matrices have been shown to support EPC differentiation and vessel formation. According to a study by Critser *et al*., EPCs vascularize collagen-based matrices *in vivo *although increasing collagen concentration significantly decreased EPC-derived vessels density but significantly increased vessel lumen sizes, suggesting that the physical properties of collagen matrices influence EPC vasculogenesis *in vivo *[115]. A more recent study described the differentiation of progenitor cells into ECs in a collagen matrix. This study showed that collagen protects progenitor cells from apoptosis and increases adhesion and invasion in an extracellular signal-regulated kinase pathway-dependent way [116].

Fibrin is also involved in the regulation of EPC biology. According to a recent study by Barsotti *et al*., when plated on fibrin, EPCs can adhere and differentiate into ECs to levels comparable to those obtained on FN, and they also show enhanced cell viability and cytokine production [100]. In another study, EPC exhibited biological activity after *in vivo *subcutaneous implantation in a fibrin matrix. Once in the fibrin matrix, EPCs migrate, differentiate into ECs and integrate into newly formed blood vessels [117]. Recent data from our laboratory further suggests that FbnE plays an essential role in EPC biology. We showed that FbnE potentiates the vasculogenic properties of EPCs and also promotes paracrine factor production. Moreover we observed that administration of a FbnE-enriched scaffold together with EPCs into mouse cutaneous wounds increases wound vascularization and consequent healing [54].

Artificial matrices include a group of scaffolds that mimic the ECM. These scaffolds combine biodegradable and biocompatible scaffolds with small synthetic peptides that mimic the integrin-binding sites of ECM molecules. Examples are synthetic mimics of FN (RGD and PHSRN) [118], laminin (Tyr-Ile-Gly-Ser-Arg motif and Ile-Lys-Val-Ala-Val motif) [119], or collagens (type I, Asp-Gly-Glu-Ala motif; type IV, Thr-Ala-Gly-Ser-Cys-Leu-Arg-Lis-Phe-Ser-Thr-Met motif) [120,121]. The FN-mimics RGD and PHSRN have both been implicated in the differentiation of cells toward the endothelial lineage [122]. A recent work by Alobaid *et *a.l showed an enhanced attachment and increased endothelial outgrowth when EPCs were cultured on RGD-coated well plates [123], while Ferreira *et al. *reported a 20-fold increase in EC differentiation from embryonic stem cells when these cells were encapsulated in a dextran-based hydrogel containing the RGD peptide [118].

The use of scaffolds mimicking the ECM in physiological processes was addressed by different investigators. Blindt and colleagues investigated this concept by incorporating integrin-binding cyclic RGD (cRGD) peptide with α_v_β_3_-integrin-binding capacities into a newly designed polymer stent-coating. This stent was analyzed in *in vitro *and *in vivo *porcine models for its potential to recruit and bind EPCs and limit coronary neointimal formation. *In vitro*, the cRGD peptide stimulated the outgrowth, shear-resistant recruitment and migration of EPCs. *In vivo*, 12 weeks after implantation, both mean neointimal area and mean percent area stenosis were significantly reduced in cRGD peptide-loaded polymer stents by accelerated stent endothelialization compared with the control stents [124]. In another study, Kim *et al. *described that targeted delivery of EPCs using an RGD-g-poly-L-lactic acid scaffold enhances dermal wound closure and vascularization in mouse models of wound healing. EPCs grown on RGD-g-poly-L-lactic acid scaffolds show increased proliferation, endothelial differentiation and consequent incorporation on wound vessels [125]. Together, these different approaches suggest targeted manipulation of ECM and/or integrin (adhesion) at vascular repair and formation sites may improve EPC function and thereby improve the neovascularization processes.

## Conclusions

**Figure 2:**
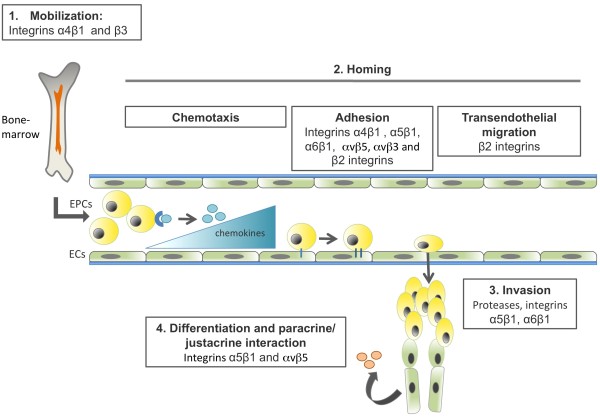
**Integrins directly involved in the multiple steps of endothelial progenitor cell biology during postnatal vasculogenesis**. Recruitment and incorporation of EPCs into angiogenic sites requires a coordinated multistep process including mobilization, chemoattraction, adhesion, endothelial transmigration, migration, tissue invasion, in situ differentiation and and/or juxtacrine factor production. The multiple integrins that have been implicated in the distinct steps of EPC biology are indicated. Figure adapted from Fusenig N, Marmé D (eds): *Tumor Angiogenesis: Basic Mechanisms and Cancer Therapy*. Springer; 2008. Chapter 17 with modifications [126].

There has been great interest in manipulating EPCs for neovascularization purposes, namely in the context of tissue regeneration and vascular repair. As is certainly clear from the abundant body of literature cited in this review, integrins play a crucial role in modulating several aspects of EPC biology and function (Figure 2). Therefore, although there has been some success in using EPCs as therapeutic agents (as shown by recent clinical trials), we suggest that targeted and tissue-specific manipulation of EPC-integrin interactions may be crucial to further improve the usage of this cell population as a relevant clinical agent.

## Abbreviations

Akt: protein kinase B; BM: bone marrow; CD: cluster of differentiation; CXCR4: CXC chemokine receptor type 4; EC: endothelial cell; ECM: extracellular matrix; EPC: endothelial progenitor cell; FN: fibronectin; FbnE: fibrin proteolytic fragment E; ICAM-1/2: inter-cellular adhesion molecule 1/2; IL: interleukin; MCP-1: monocyte chemotactic protein 1; MMP: matrix metalloproteinase; PB: peripheral blood; PHSRN: proline-histidine-serine-arginine-aspartine motif; RGD: arginine-glycine-aspartate motif; SDF-1: stromal derived factor 1; VCAM-1: vascular cell adhesion 1; VEGF: vascular endothelial growth factor; VEGFR: vascular endothelial growth factor receptor; vWF: von Willebrand factor.

## Competing interests

The authors declare that they have no competing interests.

## Authors' contributions

Both authors contributed equally to the conception of the manuscript. Both authors read and approved the final manuscript.
